# Knockdown of 11β-hydroxysteroid dehydrogenase type 1 alleviates LPS-induced myocardial dysfunction through the AMPK/SIRT1/PGC-1α pathway

**DOI:** 10.7555/JBR.36.20220212

**Published:** 2023-05-29

**Authors:** Dongmei Zhu, Lingli Luo, Hanjie Zeng, Zheng Zhang, Min Huang, Suming Zhou

**Affiliations:** Department of Geriatrics Intensive Care Unit, the First Affiliated Hospital of Nanjing Medical University, Nanjing, Jiangsu 210029, China

**Keywords:** 11β-HSD1, LPS, sepsis-induced myocardial dysfunction, inflammation, oxidative stress

## Abstract

Sepsis-induced myocardial dysfunction is primarily accompanied by severe sepsis, which is associated with high morbidity and mortality. 11β-hydroxysteroid dehydrogenase type 1 (11β-HSD1), encoded by *Hsd11b1*, is a reductase that can convert inactive cortisone into metabolically active cortisol, but the role of 11β-HSD1 in sepsis-induced myocardial dysfunction remains poorly understood. The current study aimed to investigate the effects of 11β-HSD1 on a lipopolysaccharide (LPS)-induced mouse model, in which LPS (10 mg/kg) was administered to wild-type C57BL/6J mice and 11β-HSD1 global knockout mice. We asscessed cardiac function by echocardiography, performed transmission electron microscopy and immunohistochemical staining to analyze myocardial mitochondrial injury and histological changes, and determined the levels of reactive oxygen species and biomarkers of oxidative stress. We also employed polymerase chain reaction analysis, Western blotting, and immunofluorescent staining to determine the expression of related genes and proteins. To investigate the role of 11β-HSD1 in sepsis-induced myocardial dysfunction, we used LPS to induce lentivirus-infected neonatal rat ventricular cardiomyocytes. We found that knockdown of 11β-HSD1 alleviated LPS-induced myocardial mitochondrial injury, oxidative stress, and inflammation, along with an improved myocardial function; furthermore, the depletion of 11β-HSD1 promoted the phosphorylation of adenosine 5′-monophosphate-activated protein kinase (AMPK), peroxisome proliferator-activated receptor gamma coactivator 1α (PGC-1α), and silent information regulator 1 (SIRT1) protein levels both *in vivo* and *in vitro*. Therefore, the suppression of 11β-HSD1 may be a viable strategy to improve cardiac function against endotoxemia challenges.

## Introduction

Sepsis is the leading cause of mortality among patients in intensive care units. Sepsis can result in life-threatening organ dysfunction, which is caused by the host's maladjusted response to infection^[1]^. Among patients with complications from sepsis, the heart is one of the most affected organs. The incidence of sepsis-induced myocardial dysfunction (SIMD) ranges from 10% to 70%^[2]^, which is clinically manifested by severe myocardial depression, left and right ventricular systolic and diastolic dysfunction, and reduced ejection fraction (EF). In patients with SIMD, the mortality increases from approximately 70% to 90%^[3].^ Although SIMD has gained considerable research attention, its underlying mechanism remains unknown, and there are no effective therapeutic methods or drugs to reverse it.

Elevated cortisol levels are associated with a high mortality in sepsis^[4]^. 11β-Hydroxysteroid dehydrogenase type 1 (11β-HSD1), encoded by *Hsd11b1*, is a reductase capable of converting inactive cortisone to active hydrocortisone, thus increasing local glucocorticoid levels^[5]^. Glucocorticoids can effectively regulate immune states and inflammatory responses. Patients with a higher expression of 11β-HSD1, who receive hydrocortisone, achieve a slower shock resolution than those receiving a placebo. Thus, changes in the gene expression may explain differences in response to the corticosteroid therapy during the septic shock^[6]^, although the role of 11β-HSD1 remains elusive.

Recent evidence implicates the involvement of 11β-HSD1 in cardiovascular diseases; knockout of 11β-HSD1 promotes angiogenesis in healing and improves cardiac function after myocardial infarction^[7]^. 11β-HSD1 also suppresses cardiac fibroblast neutrophil chemoattractants and neutrophil recruitment to the heart post-myocardial infarction^[8]^. Interestingly, 11β-HSD1 inhibitors can alleviate myocardial hypertrophy and the inflammatory responses induced by a high-fat diet^[9]^. However, the role of 11β-HSD1 in sepsis, and even in SIMD, remains poorly understood.

Considering the role of 11β-HSD1 in inflammation and heart disease, we speculated that 11β-HSD1 might be involved in SIMD. In the current study, we administered lipopolysaccharide (LPS) to wild-type (WT) C57BL/6J mice and 11β-HSD1 global knockout mice. The results demonstrated that the knockdown of 11β-HSD1 alleviated LPS-induced myocardial mitochondrial injury, oxidative stress, and inflammation, along with an improved myocardial function. In addition, the underlying mechanisms were also investigated.

## Materials and methods

### Experimental animals

The Laboratory Animal Ethics Committee and the Laboratory Animal Center at Nanjing Medical University granted their approval for all animal experiments in the current study (No. 2202025). Eight-week-old male 11β-HSD1 global knockout (11β-HSD1^−/−^) mice and wild-type (WT) C57BL/6J mice were procured from GemPharmatech Co., Ltd. (Nanjing, China). The mice were housed in individual cages (six mice per cage) under a climate-regulated setting (45%–50% humidity, [22.8 ± 2.0] ℃) with a 12/12-hour dark/light cycle and specific pathogen-free environment. Both food and water were easily accessible to the mice *ad libitum*. A mouse model with SIMD was established by a single-dose intraperitoneal injection of LPS (10 mg/kg, *Escherichia coli* O111:B4; Sigma-Aldrich, St. Louis, MO, USA). Mice in the control group were administered an equivalent dose of sterile saline. Based on prior findings, the time point of six hours and the specific dosage for the LPS administration were selected for the experiment^[10-11]^. After acclimatization to their surroundings, the mice were randomly divided into the following four categories: WT mice injected with sterile saline (the WT group), 11β-HSD1^−/−^ mice injected with sterile saline (the 11β-HSD1^−/−^ group), WT mice injected with LPS (the WT-LPS group), and 11β-HSD1^−/−^ mice injected with LPS (the 11β-HSD1^−/−^-LPS group). Each group comprised eight mice. Mice were intraperitoneally injected with 10 mg/kg LPS or sterile saline for six hours, and cardiac functions were evaluated. A cervical dislocation was performed to sacrifice the mice, then serum and heart tissues were collected.

### Echocardiography

The Vevo770 high-resolution *in vitro* imaging system fitted with a 35 MHz transducer (Visualsonics, Toronto, Ontario, Canada) was used to examine the heart function of mice that had been sevoflurane-anesthetized before the experiment. Measurements of the left ventricular end-diastolic inner diameter and end-systolic inner diameter were taken in the M-mode view; subsequently, the fractional shortening (FS) and the EF were derived and examined.

### Serum biochemical analysis

In preparation for further examination, serum samples were taken from each animal in various groups and frozen at −80 ℃. The levels of lactate dehydrogenase (LDH), creatine kinase isoenzyme MB (CK-MB), and creatine kinase (CK) in the serum were determined using the automatic biochemical analyzer (Chemrey240, Shenzhen, China).

### Transmission electron microscopy

After removing the hearts, small tissue blocks were extracted from the central region of the left ventricular wall and fixed in osmium tetroxide in phosphate-buffered saline (PBS, pH 7.4; HyClone, Logan, UT, USA) with 1.5% potassium ferricyanide. After dehydration using an ethanol gradient, the tissue samples were embedded with propylene oxide, an intermediary solvent. Lead citrate and uranyl acetate were used to stain ultrathin slices with thicknesses ranging from 50 to 70 nanometers. An electron microscope (H-7650, Hitachi, Tokyo, Japan) was utilized to capture and analyze the images.

### Immunohistochemistry

Each myocardial sample from the mice was fixed in formalin, embedded in paraffin, and finally sliced into 5-μm-thick sections. Subsequently, the paraffin tissue slices were soaked in a xylene solution and an ethanol gradient before deparaffinization and hydration. Afterwards, they were heated in a microwave for 23 min following immersion in a citric acid buffer at pH 6.0. The sections were removed and allowed to return to room temperature to completely retrieve the antigens. Following washing, an endogenous peroxidase inhibitor was added to the samples dropwise. The mixture was then left to react for 25 min before washing. Thereafter, the samples were incubated with anti-interleukin-1β (IL-1β) and anti-interleukin-6 (IL-6) primary antibodies (dilution 1:100; Santa Cruz Biotechnology, Santa Cruz, CA, USA) overnight in a refrigerator at 4 ℃. The samples were washed the following day thoroughly and incubated at 37 ℃ for 30 min with HRP-conjugated secondary antibodies. The process of color development was then conducted after adding drops of diaminobenzidine. Finally, after counterstaining using hematoxylin, the nuclei were mounted. The sections were observed after staining under a microscope, and the images were analyzed (Olympus, Tokyo, Japan).

### Treatment and culture of primary neonatal rat ventricular cardiomyocytes

Neonatal rat ventricular cardiomyocytes (NRCMs) were prepared according to a previously described protocol^[9]^. The hearts of 1- to 2-day-old Sprague-Dawley rats were removed and rinsed before being homogenized in PBS (HyClone). The tissues were dispersed and subjected to several incubations at 37 ℃ in PBS containing 1.0 mg/mL of type Ⅱ collagenase (Sigma-Aldrich). After centrifugation, the cell pellet was resuspended in Dulbecco's modified eagle medium (Gibco, Grand Island, NY, USA) supplemented with 10% fetal bovine serum (Gibco), 0.1 mmol/L bromodeoxyuridine (Sigma-Aldrich), 100 mg/mL streptomycin, and 100 units/mL penicillin. The dissociated cells were resuspended and incubated at 37 ℃ for 1 h. After dilution to 1×10^6^ cells per milliliter, the cells were seeded on various culture plates according to the corresponding experimental need and incubated at 37 ℃ and 5% CO_2_.

Lentiviruses expressing 11β-HSD1-specific short hairpin RNA (shRNA) (LV-HSD1 shRNA) and negative control (LV-sh NC) were synthesized by GeneChem Inc. (Shanghai, China). Following a plating period of 48 h, cells were transfected either with LV-HSD1 shRNA or LV-NC shRNA. LPS (100 ng/mL) was added to these cells and incubated for another 24 h. The following sequence in the coding region of 11β-HSD1 was used to generate the shRNA construct for 11β-HSD1 knockdown: 5′-CCGGCTCTGGGATAATCTTGAGTCACTCGAGTGACTCAAGATTATCCCAGAGTTTTTG-3′. In compliance with the specifications of the manufacturer, lentiviruses were generated and transduced using a lentiviral-based expression system (GeneChem Inc.).

After seeding NRCMs in six-well plates (1×10^5^ cells/well), cells were categorized into three following groups: (1) the sh-NC group, NRCMs infected with LV-NC shRNA without LPS treatment; (2) the sh-NC-LPS group, NRCMs infected with LV-NC shRNA with LPS treatment, and (3) the sh-HSD1-LPS group, NRCMs infected with LV-HSD1 shRNA with LPS treatment.

### Reactive oxygen species staining

To measure reactive oxygen species (ROS) levels in mouse heart tissues, frozen slides were allowed to thaw at room temperature, and the 10-μm slices were incubated with ROS staining solution (Sigma-Aldrich) for 30 min at 37 ℃ in the dark, followed by rinsing thrice in PBS. By utilizing a fluorescence microscope (Olympus), fluorescence signals were captured at an emission wavelength of 610 nm with an excitation wavelength of 535 nm. The Aipathwell program (ServiceBio, Wuhan, Hubei, China) was used to determine the average fluorescence intensity.

Following the methodology outlined previously, the levels of intracellular ROS were measured by DCFH-DA. To each well, Dulbecco's modified eagle medium with 10 μmol/L DCFH-DA (Beyotime, Shanghai, China) was added. After 30 min of incubation at 37 ℃, the plate was exposed to an environment containing 5% CO_2_ and saturating humidity. After washing thrice in PBS, the cells were resuspended in the solution. By utilizing a fluorescent enzyme label (Bio-Tek, Winooski, VT, USA), the fluorescence intensity of more than 10^4^ cells was evaluated for each sample at an emission wavelength of 525 nm with an excitation wavelength of 488 nm.

### Evaluation of malondialdehyde and superoxide dismutase concentrations

Following homogenization and centrifugation, the supernatant obtained from the heart tissues was subjected to concentration analyses for malondialdehyde (MDA) and superoxide dismutase (SOD) using the corresponding kits (Beyotime) according to the manufacturer's instructions. The bicinchoninic acid (BCA) protein assay kit (Thermo Fisher Scientific, Waltham, MA, USA) was employed to quantify protein concentrations and normalization.

NRCMs were treated as described previously. The RIPA buffer was used to lyse the cells after harvesting, and the level of MDA in the lysates was measured by using the corresponding kit (Beyotime) following the manufacturer's instructions. Thereafter, the BCA protein assay kit was utilized to ascertain the concentrations of the proteins and standardize their respective levels.

### RNA extraction and quantitative real-time PCR

Following the manufacturer's protocol for the TRIzol reagent (Invitrogen, Carlsbad, CA, USA), total RNA was isolated either from cardiac tissues or primary NRCMs. cDNA was synthesized from 2 µg total RNA with 200 units of UM-MLV reverse transcriptase (Promega, Madison, WI, USA) in a solution containing 25 U RNase inhibitor, 0.5 mmol/L deoxynucleotide triphosphate, and 0.5 μg N15 random primers, with a total reaction volume of 25 μL. Each quantitative real-time PCR (qRT-PCR) procedure was repeated thrice in a 25 μL solution containing the SYBR Green real-time PCR Master Mix (Roche, Basel, Switzerland).

The following is an outline of the PCR procedure: a preliminary stage of denaturation at 95 ℃ for 60 s, followed by 40 cycles at 95 ℃ for 15 s, 60 ℃ for 15 s, and 72 ℃ for 45 s, and the last step of extension at 80 ℃ for 5 s. PCR was performed on the Rotor Gene-3000 plate reader (Corbett Research, Sydney, Australia). To quantitatively analyze the expression levels, the 2^−ΔΔCt^ method was employed. All data were normalized to actin mRNA levels. To facilitate comparisons across various groups, the levels of expression for each gene were standardized relative to those in the control group. The Primer 5 software was used for designing the PCR primers. Primer sequences were as follows: *Hsd11b1* forward: AAGGAGCCGCACTTATCAGA and reverse: TTCAAGGCAGCGAGACACTA; and *Actb* forward: CACGATGGAGGGGCCGGACTCATC and reverse: TAAAGACCTCTATGCCAACACAGT.

### Western blotting analysis

Myocardial tissue samples and NRCMs from each group were obtained and lysed on ice with RIPA lysis solution (Beyotime). A BCA protein assay kit (Thermo Fisher Scientific) was utilized to determine the relative levels of protein in cell lysates and tissue extracts. In each group, protein samples (30 μg per lane) were separated by 10% sodium dodecyl sulfate-polyacrylamide gel electrophoresis (SDS-PAGE) and transferred onto polyvinylidene fluoride membranes (Millipore, Boston, MA, USA). Subsequently, in a solution of Tris-buffered saline and 0.1% Tween-20 (TBST; Beyotime), 5% fat-free milk (Beyotime) was dissolved and used to block the membranes for 2 h at an ambient temperature and incubated overnight at 4 ℃ with primary antibodies against 11β-HSD1 (R&D Systems, Minneapolis, MN, USA), PGC-1α (Proteintech, Chicago, IL, USA), p-AMPK, AMPK, SIRT1, GAPDH, or α-tubulin (1:1000 dilution; Cell Signaling Technology, Boston, MA, USA). Subsequently, TBST was used to rinse the membranes before incubating for 1 h with an appropriate secondary antibody (1:10 000 dilution; Abcam, Cambridge, England) at ambient temperature. An imaging system (Bio-Rad, Hercules, CA, USA) was utilized for reading the fluorescent signals. The ImageJ software (Wayne Rasband, Bethesda, MD, USA) was used to analyze the signal intensities quantitatively.

### Immunofluorescence staining

Sections of cardiac tissues from freshly frozen mice with 5 μm thickness were treated with a solution of 4% formaldehyde for 30 min, followed by treatment with 0.1% Triton X-100 for the same duration. After blocking with 3% bovine serum albumin (Sigma-Aldrich) in PBS for 30 min, the samples were incubated overnight at 4 ℃ with the appropriate primary antibodies (dilution 1:100) before the addition of Cy3-, FITC-, or Cy5-conjugated relevant secondary antibodies for staining, and followed by counterstaining with 4′,6-diamidino-2-phenylindole (DAPI, 100 ng/mL; Sigma-Aldrich) for 5 min and subsequent imaging using a fluorescence microscope (Nikon, Tokyo, Japan).

### Statistical analysis

The GraphPad Prism 9.0 software (GraphPad Software, San Diego, CA, USA) was utilized to analyze all statistical data. The results were presented as mean ± SD, and comparisons were made using the Student's *t*-test. Differences among groups were analyzed by one-way ANOVA, followed by the Tukey's test, and *P* < 0.05 was considered statistically significant.

## Results

### 11β-HSD1 was upregulated in the myocardium of LPS-induced septic mice

**Figure 1:**
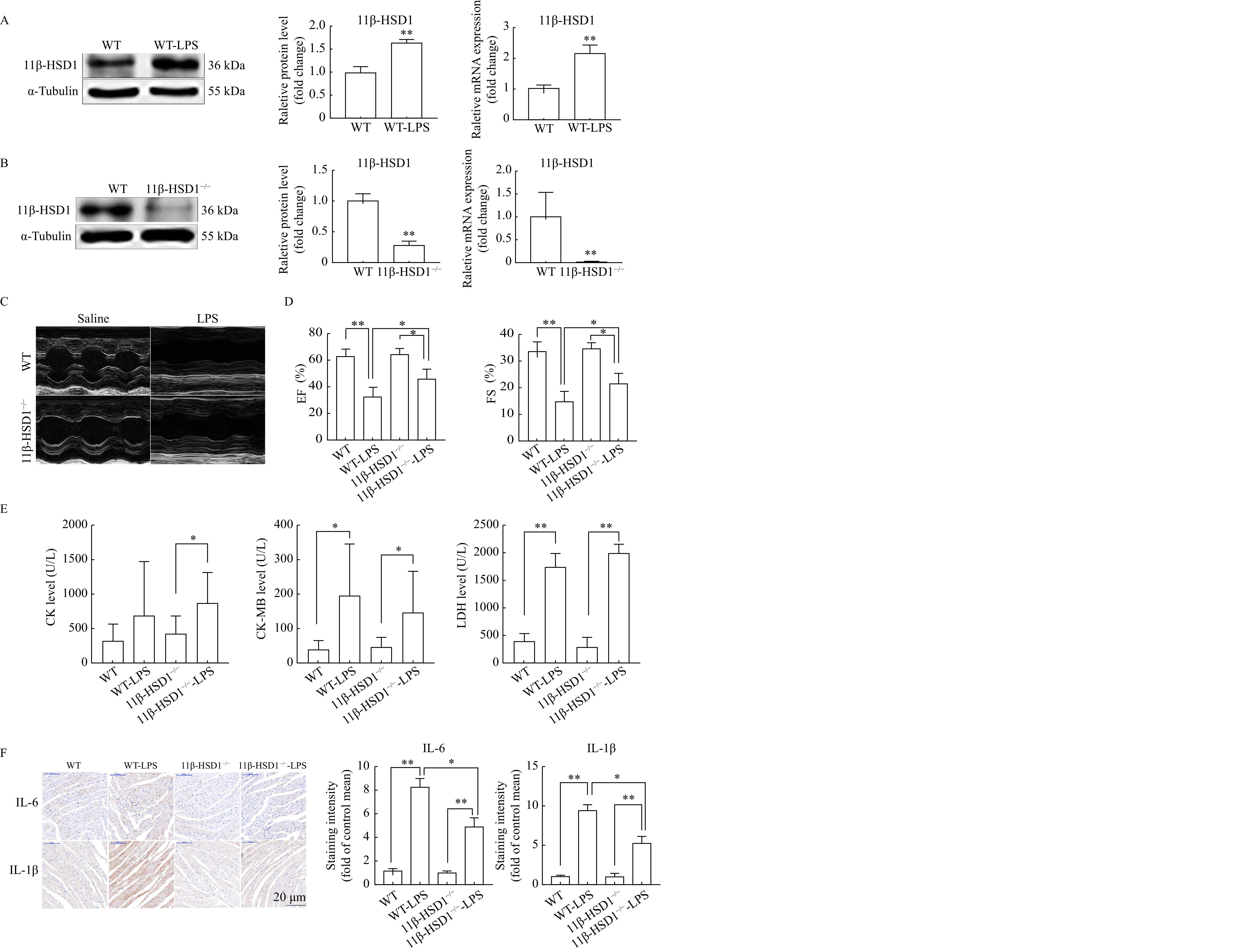
11β-HSD1 deficiency attenuated myocardial dysfunction and inflammation in LPS-induced mice.

Previous research demonstrated that pro-inflammatory cytokines like IL-1β and tumor necrosis factor-α (TNF-α) induced the upregulation of 11β-HSD1 in local tissues^[12–13]^. In the current study, mice were intraperitoneally injected with LPS at a dose of 10 mg/kg to simulate sepsis. As shown in ***Fig. 1A***, both Western blotting and qRT-PCR analyses revealed that the level of 11β-HSD1 expression in the myocardial tissue of LPS-induced mice was elevated, compared with the WT mice, suggesting that LPS stimulation increased the expression of 11β-HSD1 in the heart tissue. The protein levels of 11β-HSD1 in 11β-HSD1^−/−^ mice hearts were significantly decreased, compared with the WT mice (***Fig. 1B***).

### LPS-induced myocardial dysfunction and cardiac inflammation injury were reversed by 11β-HSD1 depletion

Mice were intraperitoneally injected with 10 mg/kg LPS to create a SIMD model. Treatment with LPS led to a significant reduction in heart function in WT mice, as indicated by the decreased EF and FS, while the knockout of 11β-HSD1 ameliorated the LPS-induced reduction in cardiac FS and EF (***Fig. 1C*** and ***1D***). As compared with the controls, the levels of CK, CK-MB, and LDH were all elevated in mice induced with LPS. No significant differences were found in cardiac levels of CK, CK-MB, and LDH between WT-LPS and 11β-HSD1^−/−^-LPS mice (***Fig. 1E***). Notably, 11β-HSD1 is a critical mediator that affects the local levels of glucocorticoids and is linked to inflammatory responses. Consequently, in the subsequent tests, the levels of inflammatory cytokines IL-1β and IL-6 were measured in the myocardium. As depicted in ***Fig. 1F***, the expression of IL-6 and IL-1β in the myocardium significantly increased after LPS administration, compared with sterile saline injection, and also decreased in the 11β-HSD1^−/−^-LPS group, compared with the WT-LPS group. These results revealed that the elimination of 11β-HSD1 attenuated LPS-induced cardiac dysfunction and cardiac inflammation, but did not significantly affect the levels of biomarkers of myocardial injury.

### Knockdown of 11β-HSD1 ameliorated mitochondrial injury and oxidative stress

**Figure 2:**
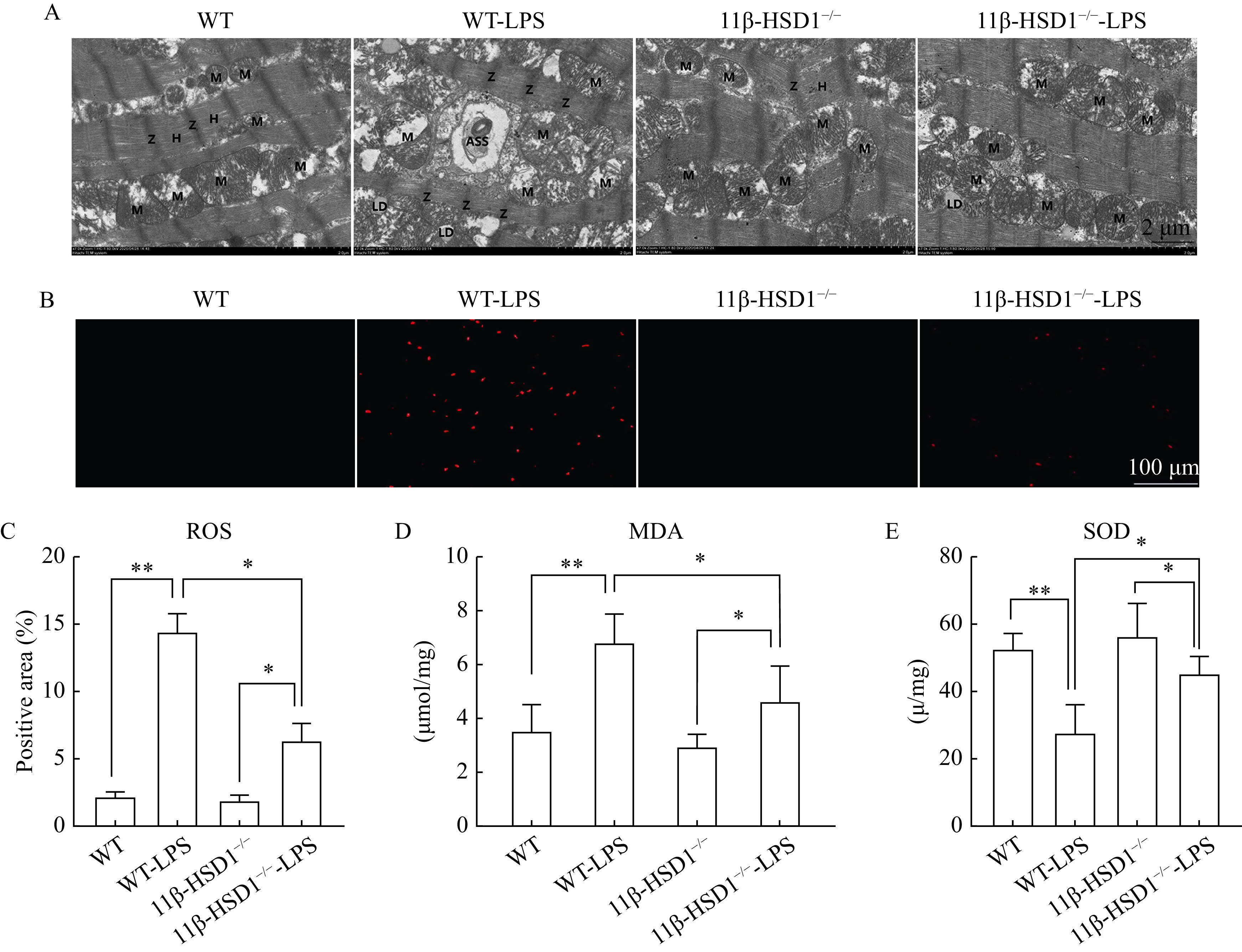
Knockdown of 11β-HSD1 ameliorates mitochondrial injury and oxidative stress.

Enhanced oxidative stress, characterized by the increased ROS levels, is a feature of sepsis. The majority of ROS in a cell originates from the mitochondria. The enzyme 11β-HSD1 has been implicated in mitochondrial dysfunction^[12,14]^. Therefore, we hypothesized that the knockout of 11β-HSD1 would affect SIMD through the mitochondria. Therefore, we conducted transmission electron microscopy analysis to visualize the morphology of mitochondria. As depicted in ***Fig. 2A***, mitochondrial swelling, ruptured cristae, and lipid droplet formation was induced following intraperitoneal injection of LPS. Mitochondrial injury improved in the 11β-HSD1^−/−^-LPS group, compared with the WT-LPS group.

Furthermore, ROS staining in myocardial tissues showed a reduced ROS production in the myocardial tissue of 11β-HSD1^−/−^ mice, compared with the LPS-treated WT mice (***Fig. 2B*** and ***2C***). We also measured MDA and SOD levels in the myocardium to assess LPS-induced oxidative stress damage. MDA is a by-product of lipid oxidation, and SOD is an antioxidant enzyme correlated with oxidative stress^[15–16]^. Both ***Fig. 2D*** and ***2E*** demonstrate that, compared with the control group, LPS led to a substantial elevation in the level of MDA and a reduction in SOD in the myocardium of WT mice. These changes dramatically improved in the 11β-HSD1^−/−^-LPS group, compared with the WT-LPS group. These findings suggest that the knockdown of 11β-HSD1 may ameliorate mitochondrial injury and oxidative stress induced by LPS administration.

### Knockdown of 11β-HSD1 attenuated oxidative stress in LPS-induced cardiomyocytes

**Figure 3:**
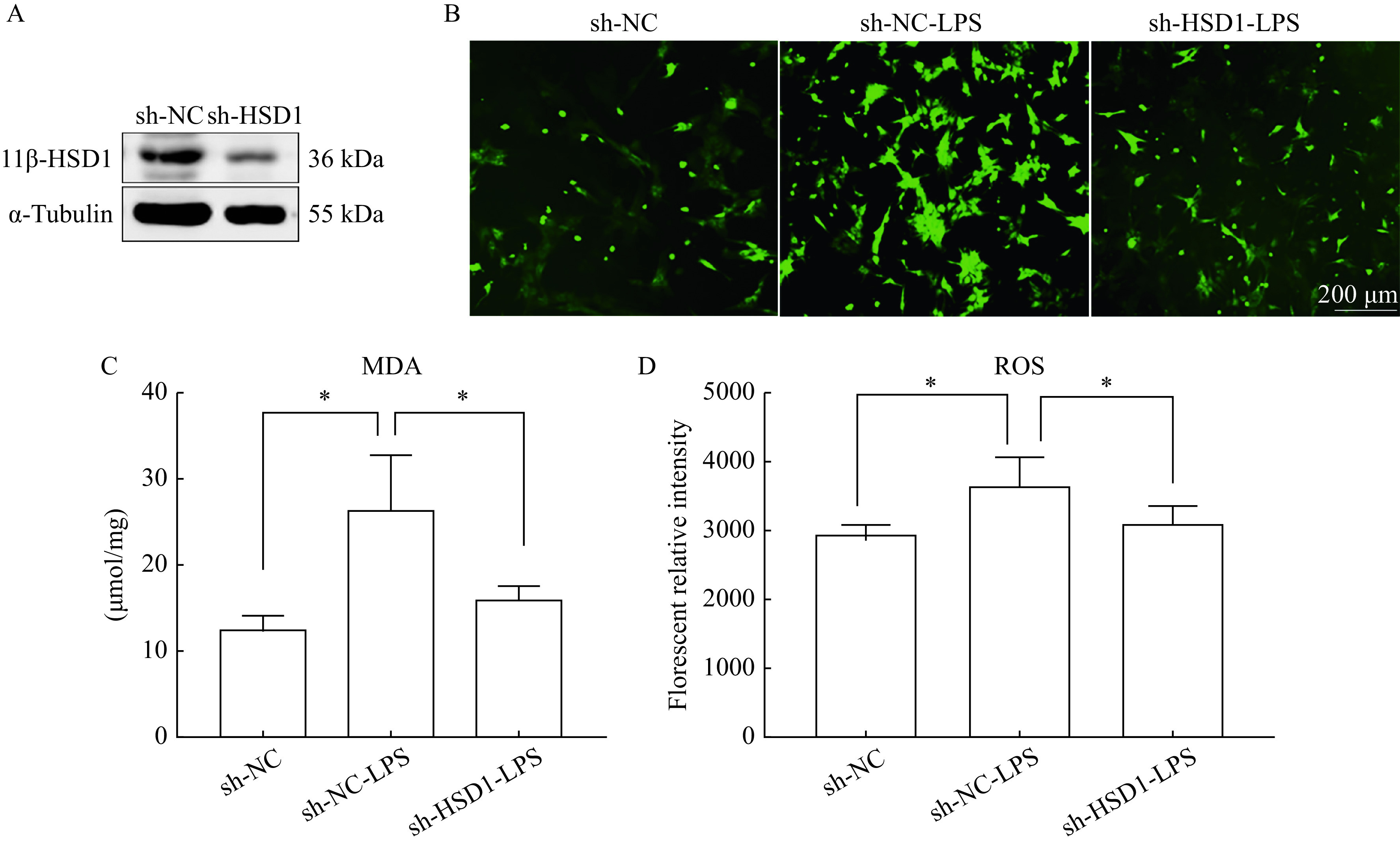
Knockdown of 11β-HSD1 attenuated oxidative stress in LPS-induced cardiomyocytes.

To examine the function of 11β-HSD1 in LPS-induced myocardial dysfunction, we used lentivirus vectors carrying the shRNA against rat 11β-HSD1 mRNA (sh-HSD1) to downregulate 11β-HSD1 expression in NRCMs. We transfected the control NRCMs with a negative control lentivirus vector (sh-NC) (***Fig. 3A***). As illustrated in ***Fig. 3B***, silencing 11β-HSD1 remarkably alleviated cardiomyocyte oxidative stress in NRCMs treated with LPS. Moreover, LPS significantly increased ROS and MDA levels in NRCMs; however, ROS and MDA levels were lower after transfection with shRNA targeting 11β-HSD1. Thus, the knockdown of 11β-HSD1 may reverse oxidative stress, thus alleviating the LPS-induced myocardial dysfunction (***Fig. 3C*** and ***3D***).

### Knockdown of 11β-HSD1 attenuated LPS-induced myocardial dysfunction* via *the activation of the AMPK/SIRT1/PGC-1α signaling pathway

**Figure 4:**
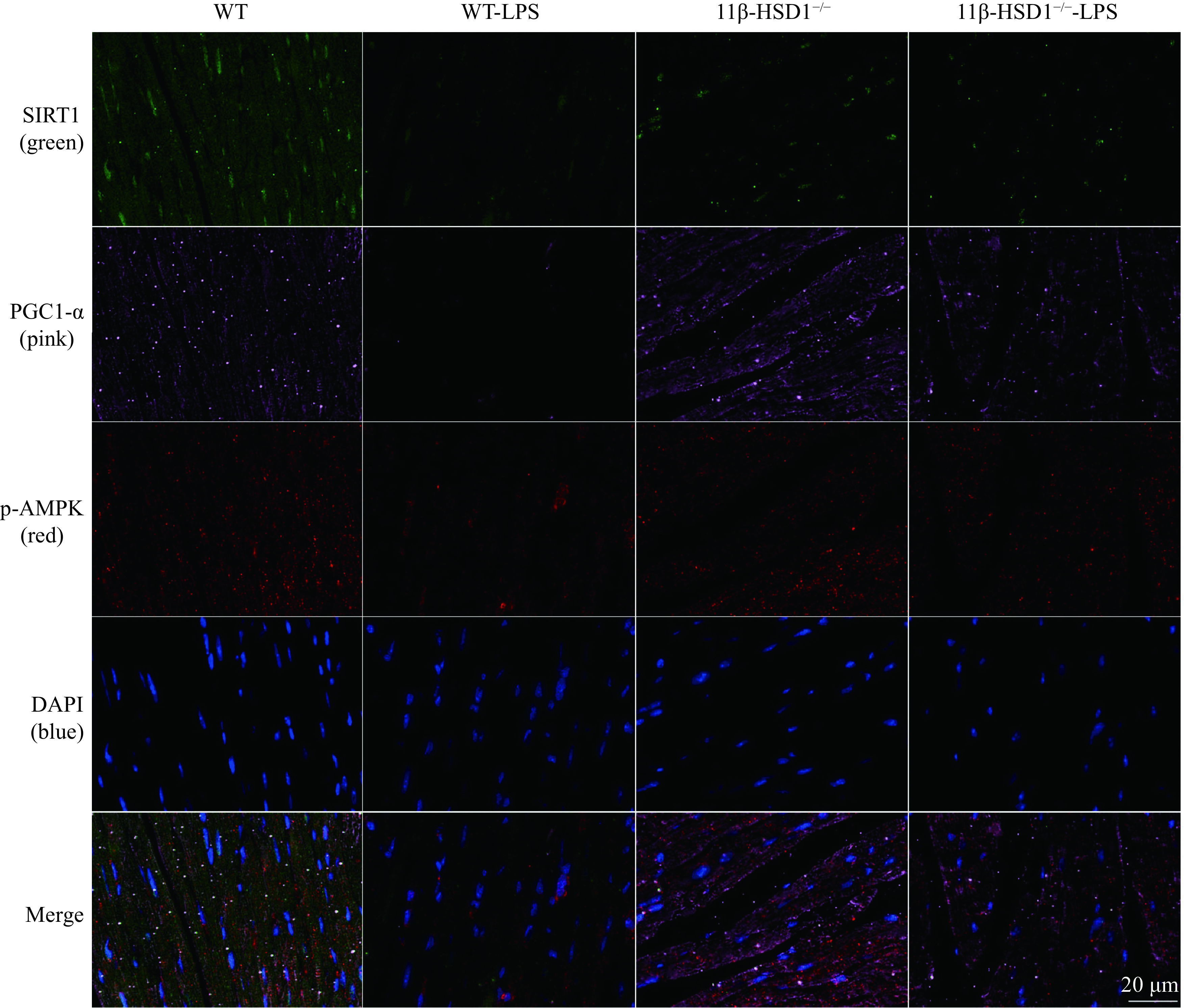
Immunofluorescence staining of myocardial tissues.

**Figure 5:**
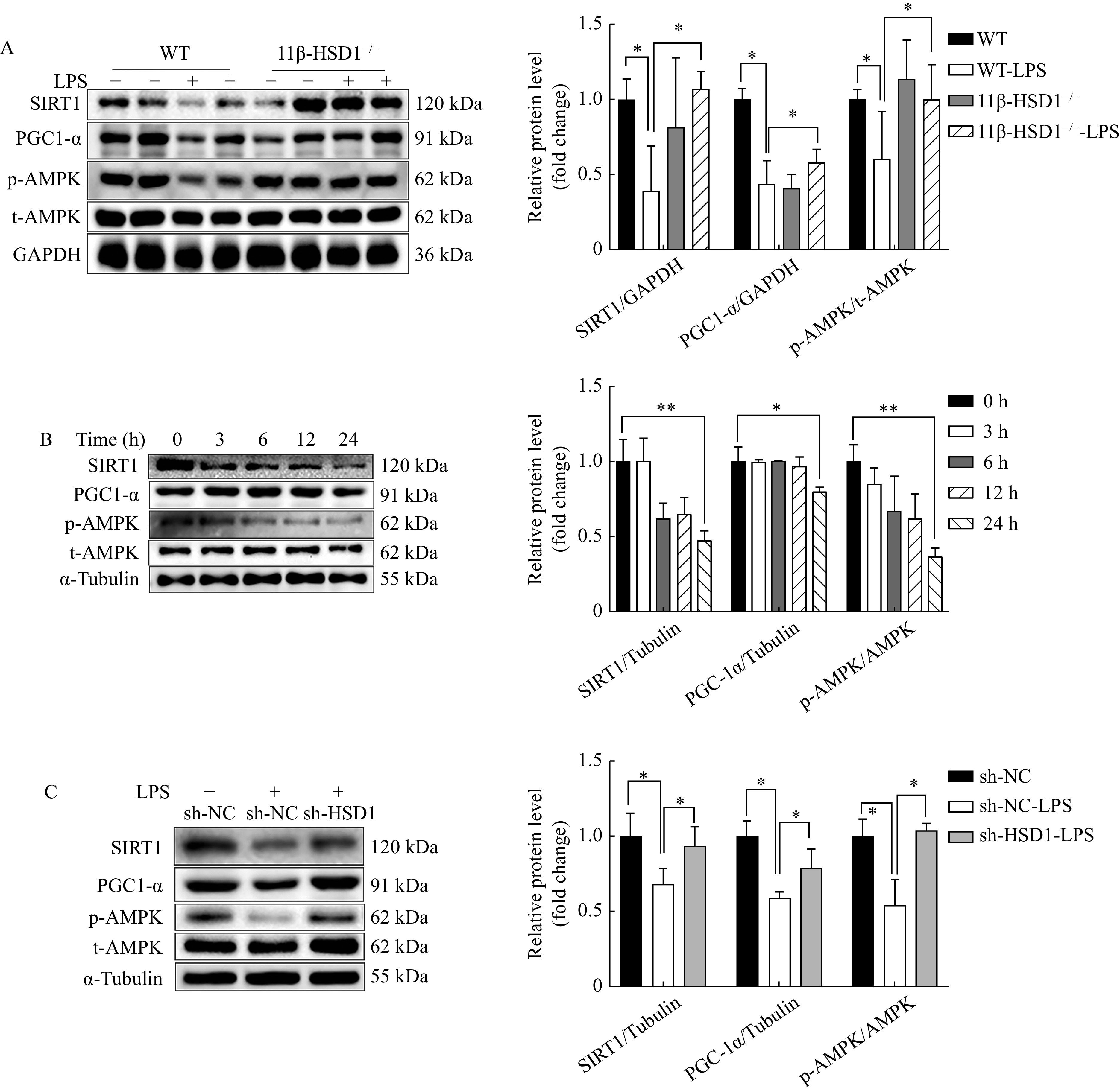
Knockdown of 11β-HSD1 activated the AMPK/SIRT1/PGC-1α signaling pathway.

An energy sensor known as adenosine 5′-monophosphate‐activated protein kinase (AMPK) is responsible for regulating cellular metabolic processes. Numerous studies have shown that AMPK has specific regulatory effects on mitochondrial biology and energy homeostasis. AMPK regulates genes encoding compositions of the mitochondrial respiratory chain and genes involved in lipid metabolism in skeletal muscle cells, thus increasing the activity of silent information regulator 1 (SIRT1) and mediating its downstream deacetylation of peroxisome proliferator-activated receptor gamma coactivator 1α (PGC-1α)^[17]^. Therefore, the AMPK/SIRT1/PGC-1α axis is an essential signaling pathway for reducing oxidative stress by modulating the expression of genes implicated in energy homeostasis and mitochondrial biosynthesis^[18]^. As shown in ***Fig. 4*** and ***Fig. 5A***, both cardiac immunofluorescence staining and protein analysis revealed that SIRT1, PGC-1α, and p-AMPK levels significantly reduced in the WT-LPS group, compared with the WT group. However, the expression of SIRT1, PGC-1α, and p-AMPK was considerably elevated in the 11β-HSD1^−/−^-LPS group, compared with the WT-LPS group, indicating that knocking down of 11β-HSD1 promoted the AMPK/SIRT1/PGC1α pathway by inhibiting 11β-HSD1 (***Fig. 5A***). Furthermore, the expression of SIRT1, PGC-1α, and p-AMPK decreased over time in NRCMs after LPS stimulation (***Fig. 5B***). However, the levels of these proteins significantly increased, following the elimination of 11β-HSD1 (***Fig. 5C***). Our results revealed that knocking down 11β-HSD1 protected against endotoxemia-induced cardiac dysfunction by attenuating oxidative stress and inflammatory responses *via* the AMPK/SIRT1/PGC-1α pathway.

## Discussion

The present study showed that intraperitoneal injection of LPS caused myocardial dysfunction, leading to a compensatory increase in 11β-HSD1 in the heart. A previous study has demonstrated that 11β-HSD1 is vital to the pathophysiology of the heart. The depletion of 11β-HSD1 or treatment with its inhibitors benefits against atherosclerosis in an animal model of acute infarction^[19]^. Our findings revealed that the knockdown of 11β-HSD1 exerted a protective effect on LPS-induced cardiac dysfunction.

Excessive inflammation is a characteristic of sepsis. Previous research has confirmed that 11β-HSD1 inhibitors attenuate LPS-induced inflammatory responses in macrophages^[20]^. Moreover, 11β-HSD1 regulates inflammatory responses, and we consistently observed that the knockdown of 11β-HSD1 lowered the expression levels of pro-inflammatory mediators (IL-6 and IL-1β) in the myocardium, suggesting a reduction in the levels of local inflammatory factors in the myocardium during endotoxemia.

Mitochondrial oxidative phosphorylation results in ROS generation. Additionally, impaired tissue perfusion, impaired oxygen metabolism, increased uncoupling, and imbalance in oxidative/antioxidant regulation in sepsis lead to a reduction in ROS clearance and an increase in ROS production, further causing damage and mitochondrial dysfunction^[21–22]^. According to previous reports, glucocorticoids are closely related to mitochondrial function. For instance, studies have shown that in brown fat, interference with 11β-HSD1 significantly increases mitochondrial content in cells and facilitates the consumption of cellular oxygen; conversely, high expression of 11β-HSD1 significantly inhibits mitochondrial function^[23]^. The 11β-HSD1 inhibitor KR-66344 decreases the production of inflammatory factors and reduces ROS generation, ultimately improving the survival of LPS-induced septic mice^[20]^. Thus, 11β-HSD1 is an essential factor for regulating the inflammatory response, oxidative stress, and mitochondrial function. The current study revealed that the knockdown of 11β-HSD1 improved LPS-induced cardiac dysfunction. Other studies have shown that the suppression of 11β-HSD1 attenuates LPS-induced changes in the morphology of the myocardial mitochondria. *In vitro* and *in vivo* studies proved that the knockdown of 11β-HSD1 reduced myocardial oxidative stress. Furthermore, the serine/threonine-protein kinase known as AMPK is an essential sensor of cellular energy metabolic processes. The decreased AMPK activity is a typical signature of cardiac dysfunction. Several reports have confirmed the protective impacts of AMPK activators on the cardiovascular system and the multifaceted effects on the regulation of mitochondrial function. In the current study, the knockdown of 11β-HSD1 enhanced myocardial AMPK phosphorylation levels and activated its downstream signaling cascade. The AMPK-regulated genes encode components of the mitochondrial respiratory chain and genes involved in lipid metabolism by increasing NAD+ levels in skeletal muscle cells, thus increasing the SIRT1 activity and regulating the downstream PGC-1α^[16]^. The AMPK/SIRT1/PGC-1α axis is a crucial pathway implicated in mitochondrial function, mediating energy metabolism, and the modulation of oxidative stress^[24]^. SIRT1 controls the expression of multiple transcriptional factors, such as PGC-1α^[25]^, which are closely associated with sepsis-induced cardiac dysfunction. The SIRT1 inhibitor EX527 reverses the protective function of nicotinamide ribosomes on sepsis-triggered cardiac injury^[26]^. Furthermore, treatment with LPS resulted in a substantial decrease in the level of SIRT1 expression in both the myocardium and the NRCMs. Notably, the levels of SIRT1 in the myocardium of LPS-treated 11β-HSD^−/−^ mice were remarkably elevated, unlike those in LPS-treated WT mice. These results suggest that the downregulation of 11β-HSD1 promoted the expression of SIRT1, which may contribute to the attenuation of LPS-induced cardiac dysfunction.

PGC-1α is a crucial component in the modulation of oxidative stress and mitochondrial biogenesis. It is instrumental in the sepsis-triggered cardiac dysfunction. In the current study, both *in vitro* and *in vivo* assays suggested that LPS intervention reduced PGC-1α expression in cardiac tissues and NRCMs. Inflammation inhibited PGC-1α expression, which is consistent with previous findings. Moreover, the elimination of 11β-HSD1 enhanced PGC-1α expression, and altogether, our findings suggested that depletion of 11β-HSD1 alleviated LPS-induced myocardial dysfunction probably by attenuating oxidative stress *via* the AMPK/SIRT1/PGC-1α pathway.

However, some limitations in the current study warrant further consideration. Firstly, the LPS-induced cardiac dysfunction model does not fully reflect the complexity and variability of clinical conditions in septic patients. Secondly, the mechanism was not further validated with relevant inhibitors or agonists, and thus further studies are needed. Thirdly, due to laboratory equipment limitations, the concentration of circulating and tissue glucocorticoids were not be detected.

**Figure 6:**
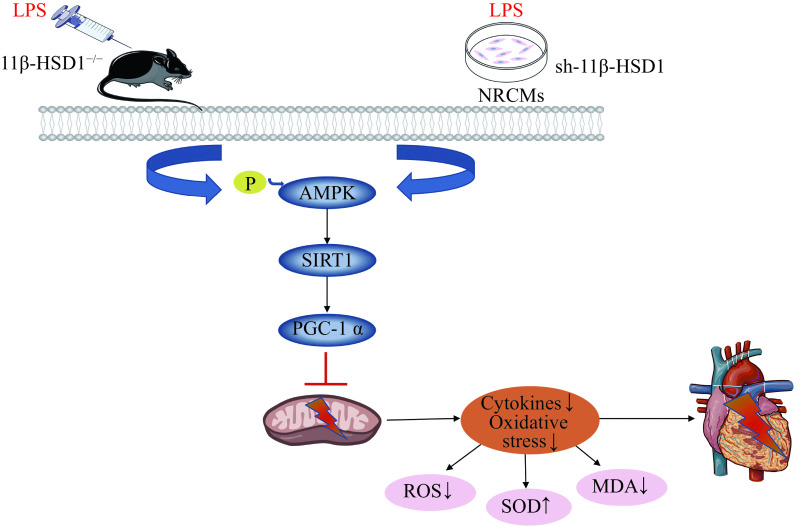
Knockdown of 11β-HSD1 alleviated LPS-induced myocardial dysfunction by attenuating inflammation and oxidative stress *via* the AMPK/SIRT1/PGC-1α pathway.

In summary, our experiments demonstrate that the knockdown of 11β-HSD1 attenuates LPS-induced myocardial dysfunction. These beneficial effects lead to the alleviation of myocardial inflammation and mitochondrial injury as well as the attenuation of oxidative stress. Furthermore, we have shown that the knockdown of 11β-HSD1 exerts protective effects by activating the AMPK/SIRT1/PGC-1α pathway (***Fig. 6***). Thertefore, targeting 11β-HSD1 may serve as a therapeutic method for preventing and treating SIMD.

## References

[1] (2021). Surviving sepsis campaign: international guidelines for management of sepsis and septic shock 2021. Crit Care Med.

[2] (2022). Pathogenetic mechanisms of septic cardiomyopathy. J Cell Physiol.

[3] (2018). Pathophysiology, echocardiographic evaluation, biomarker findings, and prognostic implications of septic cardiomyopathy: a review of the literature. Crit Care.

[4] (2019). Cortisol and adrenal androgens as independent predictors of mortality in septic patients. PLoS One.

[5] (2021). 11β-hydroxysteroid dehydrogenases: A growing multi-tasking family. Mol Cell Endocrinol.

[6] (2021). The relationship between adrenocortical candidate gene expression and clinical response to hydrocortisone in patients with septic shock. Intensive Care Med.

[7] (2010). Improved heart function follows enhanced inflammatory cell recruitment and angiogenesis in 11betaHSD1-deficient mice post-MI. Cardiovasc Res.

[8] (2017). 11β-HSD1 suppresses cardiac fibroblast CXCL2, CXCL5 and neutrophil recruitment to the heart post MI. J Endocrinol.

[9] (2018). 11β-hydroxysteroid dehydrogenase type 1 inhibitor attenuates high-fat diet induced cardiomyopathy. J Mol Cell Cardiol.

[10] (2018). Beclin-1-dependent autophagy protects the heart during sepsis. Circulation.

[11] (2019). Impaired SIRT3 activity mediates cardiac dysfunction in endotoxemia by calpain-dependent disruption of ATP synthesis. J Mol Cell Cardiol.

[12] (2016). Inhibition of 11β-hydroxysteroid dehydrogenase type 1 ameliorates obesity-related insulin resistance. Biochem Biophys Res Commun.

[13] (2012). BVT. 2733, a selective 11β-hydroxysteroid dehydrogenase type 1 inhibitor, attenuates obesity and inflammation in diet-induced obese mice. PLoS One.

[14] (2015). 11β-HSD1 reduces metabolic efficacy and adiponectin synthesis in hypertrophic adipocytes. J Endocrinol.

[15] (2017). Myocardial oxidative stress correlates with left ventricular dysfunction on strain echocardiography in a rodent model of sepsis. Intensive Care Med Exp.

[16] (2022). Oxidant/antioxidant status is impaired in sepsis and is related to anti-apoptotic, inflammatory, and innate immunity alterations. Antioxidants (Basel).

[17] (2021). AMP-activated protein kinase: a remarkable contributor to preserve a healthy heart against ROS injury. Free Radic Biol Med.

[18] (2021). Melanocortin 1 receptor attenuates early brain injury following subarachnoid hemorrhage by controlling mitochondrial metabolism *via* AMPK/SIRT1/PGC-1α pathway in rats. Theranostics.

[19] (2016). Cardiomyocyte and vascular smooth muscle-independent 11β-hydroxysteroid dehydrogenase 1 amplifies infarct expansion, hypertrophy, and the development of heart failure after myocardial infarction in male mice. Endocrinology.

[20] (2016). Anti-inflammatory effect of a selective 11β-hydroxysteroid dehydrogenase type 1 inhibitor via the stimulation of heme oxygenase-1 in LPS-activated mice and J774.1 murine macrophages. J Pharmacol Sci.

[21] (2017). Clarifying the supercomplex: the higher-order organization of the mitochondrial electron transport chain. Nat Struct Mol Biol.

[22] (2019). Mitochondrial bioenergetics links inflammation and cardiac contractility in endotoxemia. Basic Res Cardiol.

[23] (2013). Essential roles of 11β-HSD1 in regulating brown adipocyte function. J Mol Endocrinol.

[24] (2020). PGC-1*α*, inflammation, and oxidative stress: an integrative view in metabolism. Oxid Med Cell Longev.

[25] (2015). Mitochondrial biogenesis is impaired in osteoarthritis chondrocytes but reversible via peroxisome proliferator-activated receptor γ coactivator 1α. Arthritis Rheumatol.

[26] (2018). Administration of nicotinamide riboside prevents oxidative stress and organ injury in sepsis. Free Radic Biol Med.

